# Hepatic steatosis and steatohepatitis: a functional meta-analysis of sex-based differences in transcriptomic studies

**DOI:** 10.1186/s13293-021-00368-1

**Published:** 2021-03-25

**Authors:** José F. Català-Senent, Marta R. Hidalgo, Marina Berenguer, Gopanandan Parthasarathy, Harmeet Malhi, Pablo Malmierca-Merlo, María de la Iglesia-Vayá, Francisco García-García

**Affiliations:** 1 Bioinformatics and Biostatistics Unit, Principe Felipe Research Center, Valencia, Spain; 2 Spanish National Bioinformatics Institute, ELIXIR-Spain (INB, ELIXIR-ES), Madrid, Spain; 3 Liver Transplantation and Hepatology Unit, Hospital Universitario y Politécnico La Fe, Valencia, Spain; 4 Grupo de Hepatología, Cirugía HBP y Trasplantes, Instituto de Investigación Sanitaria La Fe, Valencia, Spain; 5 CIBERehd, Centro de Investigación Biomédica en Red en Enfermedades Hepáticas y Digestivas, Instituto de Salud Carlos III, Madrid, Spain; 6 Department of Medicine, Universitat de València, Valencia, Spain; 7 Division of Gastroenterology and Hepatology, Mayo Clinic, Rochester, MN USA; 8 Atos Research & Innovation (ARI), Madrid, Spain; 9 Biomedical Imaging Unit FISABIO-CIPF, Fundación para el Fomento de la Investigación Sanitario y Biomédica de la Comunidad Valenciana, Valencia, Spain

**Keywords:** Non-alcoholic fatty liver disease, Sex characteristics, Precision medicine, Computational biology, Transcriptome profiling

## Abstract

**Background:**

Previous studies have described sex-based differences in the epidemiological and clinical patterns of non-alcoholic fatty liver disease (NAFLD); however, we understand relatively little regarding the underlying molecular mechanisms. Herein, we present the first systematic review and meta-analysis of NAFLD transcriptomic studies to identify sex-based differences in the molecular mechanisms involved during the steatosis (NAFL) and steatohepatitis (NASH) stages of the disease.

**Methods:**

Transcriptomic studies in the Gene Expression Omnibus database were systematically reviewed following the PRISMA statement guidelines. For each study, NAFL and NASH in premenopausal women and men were compared using a dual strategy: gene-set analysis and pathway activity analysis. Finally, the functional results of all studies were integrated into a meta-analysis.

**Results:**

We reviewed a total of 114 abstracts and analyzed seven studies that included 323 eligible patients. The meta-analyses identified significantly altered molecular mechanisms between premenopausal women and men, including the overrepresentation of genes associated with DNA regulation, vinculin binding, interleukin-2 responses, negative regulation of neuronal death, and the transport of ions and cations in premenopausal women. In men, we discovered the overrepresentation of genes associated with the negative regulation of interleukin-6 and the establishment of planar polarity involved in neural tube closure.

**Conclusions:**

Our meta-analysis of transcriptomic data provides a powerful approach to identify sex-based differences in NAFLD. We detected differences in relevant biological functions and molecular terms between premenopausal women and men. Differences in immune responsiveness between men and premenopausal women with NAFLD suggest that women possess a more immune tolerant milieu, while men display an impaired liver regenerative response.

**Graphical abstract:**

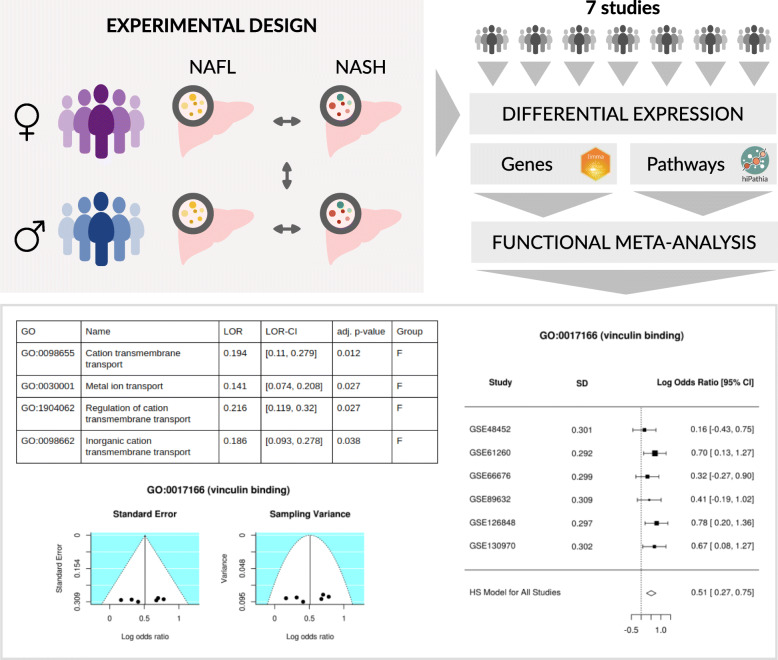

**Supplementary Information:**

The online version contains supplementary material available at 10.1186/s13293-021-00368-1.

## Background

Non-alcoholic fatty liver disease (NAFLD) encompasses a spectrum of liver disorders—ranging from fat accumulation in hepatocytes (NAFL) to non-alcoholic steatohepatitis (NASH)—which can lead to the development of cirrhosis or liver cancer. While steatosis characterizes NAFL, NASH exhibits the additional histological features of inflammation, ballooning, and fibrosis. Awareness of NAFLD has recently increased due to its worldwide impact on health; indeed, NAFLD now represents the most common liver disease in developed countries [1]. While the estimated worldwide prevalence of NAFLD is around 25%, it is more common in South America and the Middle East and less prevalent in Africa [2].

Of note, there exists significant interindividual heterogeneity regarding NAFLD progression, which has a considerable impact on the clinical consequences to the individual. Patients in the initial stage of the disease (NAFL) display a low risk of adverse outcomes [3]; however, progression to NASH increases the possibility of both hepatic and extrahepatic complications.

Interestingly, NAFLD has a higher prevalence in men and postmenopausal women than premenopausal women [4]. Indeed, the liver shows the second-largest amount of sexual dimorphism in humans [5], with both physiological and pathological hepatic processes, such as the detoxifying metabolism of cholesterol and the prevalence of hepatic diseases, respectively, differing between men and women [6]. Therefore, patient sex will likely affect NAFLD progression and treatment outcomes [7]; however, most NAFLD studies do not consider differences between sexes in their analysis [8]. We sought to explore the molecular mechanisms underpinning the likely existence of sex-related differences in NAFLD patients for the above-mentioned reasons.

In this context, we carried out the first (to the best of our knowledge) systematic review and meta-analysis of transcriptomic studies in NAFLD. We selected studies that had deposited data in the Gene Expression Omnibus (GEO) datasets database [9] to guarantee the eligibility of selected studies. We used these analyses to clarify the molecular mechanisms underpinning differences in NAFL and NASH in premenopausal women and men (postmenopausal women were not included in the analysis due to the overall low number of cases). Overall, sex-based differences may have important clinical implications for patients, and this knowledge may help to develop personalized approaches to NAFLD management.

## Materials and methods

### Literature review

A review was conducted in June 2020 following the Preferred Reporting Items for Systematic Reviews and Meta-Analyses (PRISMA) statement guidelines [10]. The GEO datasets database was searched [9] using the keywords “NAFLD”, “NAFL”, “steatosis”, “NASH”, and “steatohepatitis” for transcriptomic studies published in English.

### Study exclusion criteria

The following exclusion criteria were applied: (i) studies conducted in organisms other than humans, (ii) studies without information regarding the sex of participants or that did not include both sexes, (iii) studies without individuals from the NAFL and NASH stages of the disease, and (iv) studies in which the disease had not been diagnosed with a biopsy. In the latter case, the requirement for NAFL and NASH diagnoses by biopsy was added as less invasive methods (such as conventional imaging techniques) cannot accurately detect mild steatosis or differentiate NAFL from NASH [11]. This step aimed to minimize the input of false-positive data into the statistical analyses caused by an incorrect disease stage classification.

### Bioinformatics analysis

**Fig. 1:**
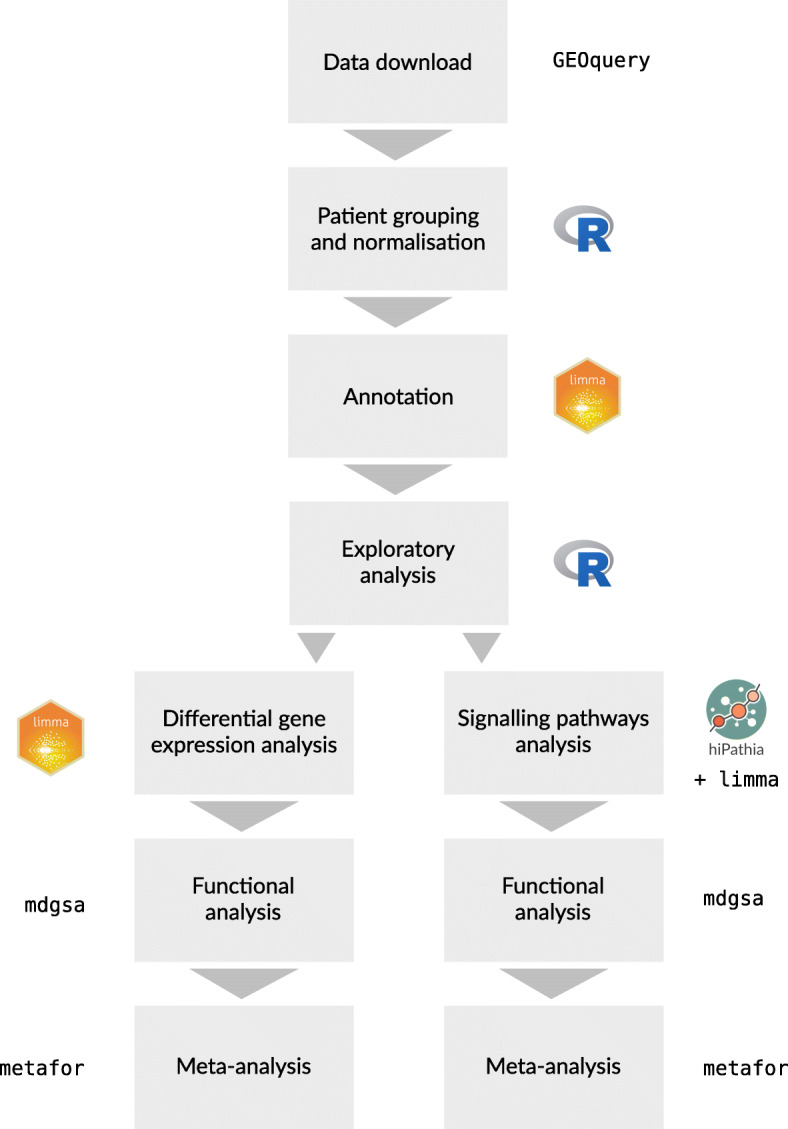
Data analysis workflow

The same three-step transcriptomic analysis strategy was applied to each separate study: (i) data acquisition and preprocessing, (ii) differential gene expression and functional enrichment analysis, and (iii) differential pathway activation and functional analysis. The functional results of all studies were integrated using meta-analytical techniques (Fig. 1). Bioinformatics analysis was carried out using the programming language R 3.6.0 [12]; information regarding the packages used and their version numbers are provided in the supplementary material.

### Data acquisition and preprocessing

During data preprocessing, the nomenclature of the distinct disease stages (by grouping patients into Control, NAFL, and NASH groups) and the association of the probe identifiers with their corresponding genes were standardized. In those studies containing treated and untreated patients, treated patients were eliminated from further analysis. For studies that used microarrays, probe codes were transformed to their respective Entrez identifiers from the NCBI database. For repeated probes, the median of their expression values was calculated. When available, data normalized by the original authors of the studies were used. Otherwise (studies GSE61260 and GSE66676), raw data were normalized using the Robust Multichip Average (RMA) algorithm [13]. The gene nomenclature in RNA-Seq studies was also standardized to the Entrez identifiers, and the raw data matrix was processed using the TMM standardization method [14] followed by a log transformation of the data.

Women were separated into premenopausal and postmenopausal groups to meet the objective of this work. As selected studies failed to include this specific information, women aged under 50 years were assumed as premenopausal based on studies indicating an average age of menopause at around 48–52 years [15, 16].

After data normalization, the detection of possible anomalous effects within the studies was carried out by completing an exploratory clustering and a principal component analysis (PCA).

### Differential gene expression and functional enrichment analysis

Differential expression analyses of selected studies were carried out using the *limma* and *edgeR* packages [17, 18] to detect differentially expressed genes when comparing NAFL and NASH in premenopausal women versus men. A linear model was adjusted for each gene, which included possible batch effects, contrasting (NASH.W – NAFL.W) and (NASH.M – NAFL.M), where NASH.W, NAFL.W, NASH.M, and NAFL.M corresponded to NASH-affected premenopausal women, NAFL-affected premenopausal women, NASH-affected men, and NAFL-affected men, respectively. Statistics regarding the differential expression were calculated and *p* values adjusted using the Benjamini & Hochberg (BH) method [19].

Based on the differential gene expression results, functional enrichment analysis was then performed using gene set analysis (GSA) [20]. First, genes were ordered according to their *p* value and the sign of the contrast statistic. Next, GSA was performed using the logistic regression model implemented in the *mdgsa* R package [21] along with their corresponding functional annotations obtained from the Gene Ontology (GO) [22] and Kyoto Encyclopedia of Genes and Genomes (KEGG) PATHWAY [23] databases.

Due to their hierarchical structure, the gene annotations with GO terms applied in the *mdgsa* package were propagated to inherit the annotations of the ancestor terms. Excessively specific or generic annotations (blocks smaller than ten or larger than 500 words) were subsequently filtered out. Finally, functions with a BH-adjusted *p* value under 0.05 were considered significant.

For the two functional elements (GO terms and KEGG paths), the number of overrepresented elements shared by the studies were analyzed. These results were graphically represented as UpSet plots [24] to demonstrate the common elements between the different sets. First, the overrepresented elements were compared as separate graphs to detect the common functions in each group: GO terms in premenopausal women, GO terms in men, KEGG pathways in premenopausal women, and KEGG pathways in men. Next, any significant GO terms in each study were visualized in the same plot to highlight significant terms with different signs among the studies.

### Differential pathway activation and functional analysis

The *hipathia* algorithm [25] was used to perform Pathway Activity Analysis (or PAA). This method transforms gene expression values for stimulus–response signaling sub-pathways into activation levels that ultimately trigger cellular responses. In this current study, the *hipathia* R package was used to analyze 1654 GO terms and 142 UniProt functions [26] associated with 146 KEGG routes [23]. The activation signal of each sub-pathway was computed from gene expression values using this algorithm. These values were used to detect significant differential activations in the (NASH.W – NAFL.W) – (NASH.M – NAFL.M) contrast pair (as defined above). The functional annotations included in *hipathia* and the differential activation results were integrated using the GSA method to identify differences at the functional level (GO terms and UniProt functions) in relation to NAFLD stages.

### Meta-analysis

Once the gene functional analysis was applied to each study, a functional meta-analysis was carried out to summarize the results. Similarly, once the pathway functional analysis was applied to each study, the results were summarized via a homologous functional meta-analysis.

Both meta-analyses were performed following the methodology described in detail by García-García [27]. Briefly, the *metafor* R package [28] was used to evaluate the combined effect within a random-effects model, which more precisely detects overrepresented elements when compared with performing studies individually at higher statistical power. Likewise, variability in the individual studies was considered in the global estimation of the measured effect so that less-variable results had a higher weight in the overall calculation of the logarithm of the odds ratio (LOR) [29]. Incorporating the variability between experiments in the model of random effects provides more statistically robust results and better integration of selected studies. Finally, the suitability of this selection was confirmed by evaluating the heterogeneity of each study in the global model and the application of cross-validation techniques.

For each meta-analysis and function, *p* values (adjusted using the BH method), LORs, and 95% confidence intervals (CIs) were calculated. A term was considered significant when its adjusted *p* value was lower than 0.05; a positive LOR in significant functions indicated a greater overrepresentation in premenopausal women than in men. In contrast, a negative LOR indicated higher representation in men than in premenopausal women. Finally, funnel plots and forest plots were used to assess the variability and measure each study’s contribution to the meta-analysis.

A total of 8223 elements were analyzed in the gene functional meta-analysis (7994 GO terms and 229 KEGG pathways); 1654 GO terms and 142 UniProt functions [26] were assessed in the functional pathway meta-analysis in association with 146 KEGG pathways [23].

### Availability of data and materials

The large volume of data and results generated in this study is freely available through the metafun-NAFLD platform: https://bioinfo.cipf.es/metafun-NAFLD. Access allows any user to analyze the results shown in this manuscript and review other results that may be of interest. The front-end was developed using the Bootstrap library. All graphics used in this tool were implemented with Plot.ly except for the exploratory analysis cluster plot, which was generated with the ggplot2 package.

In this study, transcriptomic data from the Gene Expression Omnibus database with accession numbers GSE48452, GSE61260, GSE66676, GSE83452, GSE89632, GSE126848, and GSE130970 were analyzed.

## Results

### Systematic review and exploratory analysis

**Fig. 2:**
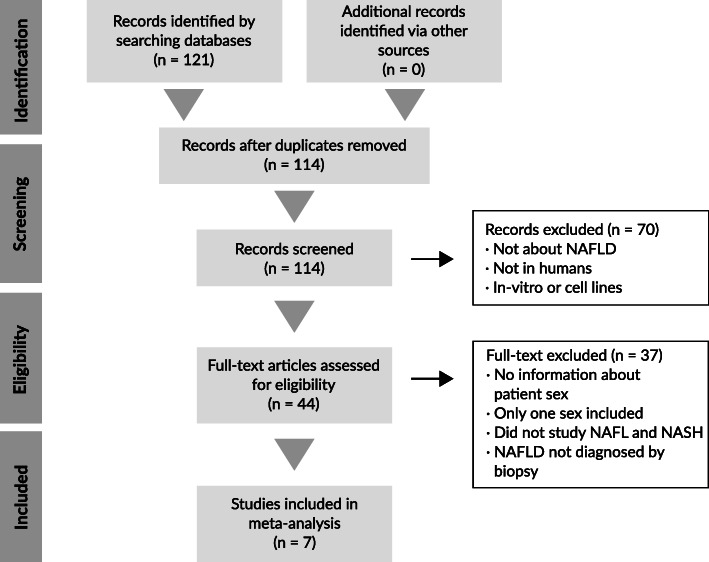
Flow diagram of our systematic review of the literature and selection of studies for meta-analysis—according to the PRISMA statement guidelines

As shown in the PRISMA flow diagram (Fig. 2), our systematic review yielded 114 non-duplicated studies. After applying the previously described exclusion criteria, we employed a total of seven studies in our overall analysis. We excluded studies for the following reasons: studies not in humans or unrelated to NAFLD (*n* = 55), in vitro studies (*n* = 15), lack of information regarding sex or inclusion of only one sex (*n* = 24), no patients in NAFL and NASH disease stages (*n* = 12), and diagnosis not based on histology (*n* = 1). The strict nature of these inclusion and exclusion criteria allowed the selection of comparable studies to ensure the reliability of the subsequent analytical strategies.

The seven selected studies [30–36] included a total of 323 eligible patients (Table 1); 164 individuals were men and 159 women under the age of 50 (49.2% men and 50.8% women, respectively). By disease stage, 148 belonged to the NAFL group and 175 to the NASH group (45.8 and 54.2%, respectively).

**Table 1:** Characteristics of the studies selected for inclusion in our analysis in terms of disease stage, sex, BMI, and patient age

GEO accession, Authors, Year [Ref.]	Patients by disease stage and sex				Age	BMI
	Premenopausal women		Men		Mean + SD	Mean + SD
	NAFL	NASH	NAFL	NASH		
GSE48452 / Ahrens M. et al. 2013 [30]	10	9	4	4	41.62 ± 9.35	47.18 ± 11.34
GSE61260 / Horvath S. et al*.* 2014 [31]	10	7	12	12	41.51 ± 9.09	51.55 ± 10.40
GSE66676 / Xanthakos S.A. *et al.* 2015 [32]	20	4	6	3	17.14 ± 1.66	53.55 ± 8.86
GSE83452 / Lefebvre P. et al*.* 2017 [33]	32	35	6	46	39.61 ± 11.51	N/D (> 25 or obese)
GSE89632 / Arendt B.M. et al. 2015 [34]	2	4	14	9	39.55 ± 8.19	30.21 ± 5.51
GSE126848 / Suppli M.P. et al*.* 2019 [35]	6	4	9	12	40 ± 13^a^	N/D (> 30)
GSE130970 / Hoang S.A. et al*.* 2019 [36]	4	12	13	14	44.72 ± 9.80	N/D

^a^The information corresponding to the patients in this study was kindly provided by the author of the study, although individual patient ages were not available

As previously indicated, we only used data from non-treated individuals in study GSE83452. Table 1 demonstrates the number of individuals from each study eligible for inclusion based on our aforementioned criteria. In an exploratory analysis, PCA of the GSE83452 study demonstrated that samples clustered into two groups. Further analysis of this study identified technical factors as the source of this grouping rather than any other clinical characteristic, as samples grouped according to their order (the first 64 samples grouped into one cluster and the last 88 into another). We considered this a batch effect, which we accounted for in our subsequent analyses.

### Individual analysis

**Fig. 3:**
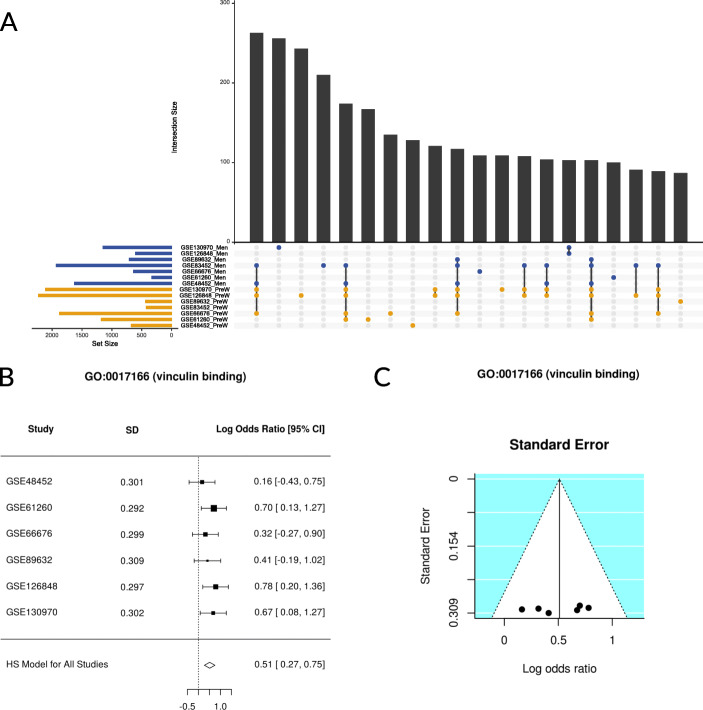
Representation of results of the bioinformatics analysis. **a** UpSet plot showing the number of common results throughout individual studies in our functional gene analysis. Only the twenty most abundant interactions are shown. Horizontal bars indicate the number of significant elements in each study (premenopausal [orange] women and men [blue]). The vertical bars indicate the common elements in the sets, indicated with dots under each bar. The single points represent the number of unique elements in each group. Those vertical bars with dots of both colors beneath indicate significant amounts of molecular bases in men and women, in a contradictory way, in different studies. **b** Example of a forest plot from the meta-analysis. This representation allows us to observe the LOR and confidence interval of the GO terms in each study (horizontal bars) and the meta-analysis (diamond). **c** Example of a funnel plot from the meta-analysis. Dots in the white area indicate the absence of bias and heterogeneity

The functional analysis of individual genes highlighted significant results overrepresented in both the groups of premenopausal women and men (summarized in Table 2). Analysis (using UpSet plots) of the common terms or pathways in the different studies demonstrated no common element in more than four studies (Supplementary Figure 1); in fact, we discovered opposite results for some elements in different studies (Fig. 3a). The functional analysis of individual pathways only exhibited significant results for the GSE83452 study (20 sub-paths and 96 GO terms).

**Table 2:** Number of significant GO terms and KEGG pathways in individual studies after gene functional analysis. Positive and negative logarithms of the odds ratio (LORs) demonstrate overrepresentation in premenopausal women and men, respectively

	GO terms		KEGG pathways	
	Positive LOR (Pre. women)	Negative LOR (Men) (Men)	Positive LOR (Pre. women)	Negative LOR (Men)
**GSE48452**	166	642	5	22
**GSE61260**	249	35	5	6
**GSE66676**	721	168	30	2
**GSE83452**	90	931	2	25
**GSE89632**	52	67	2	1
**GSE126848**	1168	150	55	5
**GSE130970**	1244	407	31	20

### Meta-analysis

We performed separate meta-analyses using the results of the two previous approaches to obtain a measure of the overall effect of each of the 8223 elements evaluated (GO terms, KEGG pathways, or UniProt functions) throughout all individual studies, i.e., the LOR and its confidence interval. This effect indicated the magnitude of the overexpression of each term (the LOR value) and its direction (the LOR sign). This sign also helped to identify the group in which the element is overrepresented. In our analysis, positive signs indicate overrepresentation in premenopausal women and negative signs as overrepresentation in men.

Along with the LOR, the meta-analysis also provides an adjusted *p* value (FDR) that indicates the significance of the results. As a result of our meta-analysis, we identified thirteen significant GO terms (adjusted *p* value < 0.05), which refer to the molecular mechanisms altered in our comparison (detailed in the following sections and Table 3). Figure 3b shows, for one function, the LOR of each study and the joint LOR of the meta-analysis. Supplementary Figures 2 and 3 detail the results of all significant elements. We confirmed the absence of bias and heterogeneity in all cases, as in Fig. 3c (Supplementary Figures 4 and 5).

**Table 3:** Significant functions in the functional meta-analyses. The logarithm of the odds ratio (LOR), its confidence interval (LOR-CI), the adjusted *p* value, and the group in which the function was overexpressed are shown (*W*, women; *M*, men)

	GO ID	Name	LOR	LOR-CI	Adj. ***p*** value	Group
**Gene functional meta-analysis**	**GO:0001012**	RNA polymerase II regulatory region DNA binding	0.132	[0.079, 0.185]	0.008	W
	**GO:1900165**	Negative regulation of interleukin-6 secretion	-0.762	[-1.088, -0.437]	0.018	M
	**GO:0000977**	RNA polymerase II regulatory region sequence-specific DNA binding	0.131	[0.073, 0.188]	0.02	W
	**GO:0017166**	Vinculin binding	0.511	[0.271, 0.75]	0.035	W
	**GO:0071352**	Cellular response to interleukin-2	0.467	[0.248, 0.687]	0.035	W
	**GO:0090177**	Establishment of planar polarity involved in neural tube closure	-0.514	[-0.748, -0.279]	0.035	M
	**GO:0090178**	Regulation of establishment of planar polarity involved in neural tube closure	-0.523	[-0.768, -0.279]	0.035	M
	**GO:1901215**	Negative regulation of neuron death	0.116	[0.06, 0.171]	0.043	W
	**GO:0070669**	Response to interleukin-2	0.446	[0.23, 0.662]	0.046	W
**Pathway functional meta-analysis**	**GO:0098655**	Cation transmembrane transport	0.194	[0.11, 0.279]	0.012	W
	**GO:0030001**	Metal ion transport	0.141	[0.074, 0.208]	0.027	W
	**GO:1904062**	Regulation of cation transmembrane transport	0.216	[0.119, 0.32]	0.027	W
	**GO:0098662**	Inorganic cation transmembrane transport	0.186	[0.093, 0.278]	0.038	W

#### Gene functional meta-analysis

**Fig. 4:**
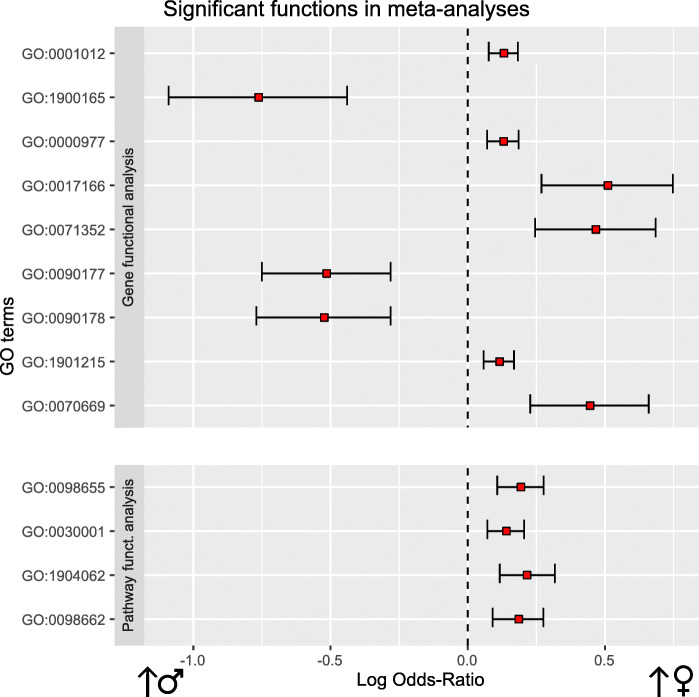
Differential functional profiling by sex. This plot shows significant functional terms of each meta-analysis. On the right: biological functions over-represented in women. On the left: biological functions more overrepresented in men. For each function, the LOR (red square) and confidence interval have been represented

The functional meta-analyses of the individual gene differential expression pipeline results provided a total of nine significant GO terms (Table 3 and Fig. 4) but no significant KEGG pathways. Among the significant GO terms, we identified six overrepresented in premenopausal women; these elements encompassed a broad spectrum of biological processes and molecular functions, including “*RNA polymerase II regulatory region DNA binding”* (GO:0001012), “*RNA polymerase II regulatory region sequence-specific DNA binding”* (GO:0000977), “*Vinculin binding”* (GO:0017166), “*Cellular response to interleukin-2”* (GO:0071352), “*Negative regulation of neuron death”* (GO:1901215), and “*Response to interleukin-2”* (GO:0070669). We identified three terms overrepresented in men: “*Negative regulation of interleukin-6 secretion”* (GO:1900165), “*Establishment of planar polarity involved in neural tube closure”* (GO:0090177), and “*Regulation of establishment of planar polarity involved in neural tube closure”* (GO:0090178).

#### Pathway functional meta-analysis

The functional meta-analysis of the individual results in the pathway differential activation pipeline returned four significant GO terms. These terms were overrepresented in premenopausal women compared with men, with a LOR value lower than 0.25 (Table 3 and Fig. 4). These terms relate to the transport of ions and cations: “*Cation transmembrane transport”* (GO:0098655), “*Metal ion transport”* (GO:0030001), “*Regulation of cation transmembrane transport”* (GO:1904062), and “*Inorganic cation transmembrane transport”* (GO:0098662). The meta-analysis of the UniProt functions failed to provide significant results.

#### Metafun-NAFLD platform

To contribute to data sharing and support the development of subsequent related studies, we generated the Metafun-NAFLD platform (https://bioinfo.cipf.es/metafun-NAFD). This web tool contains the detailed results of all the steps of the bioinformatics pipeline and allows simple interactive exploration by any interested party.

## Discussion

Although previous studies have analyzed pathobiological mechanisms in NAFLD and (separately) sexual differences in metabolic regulation, the impact of sex on NAFLD pathobiology remains incompletely defined. Epidemiological studies have reported a higher prevalence of NAFLD in men and postmenopausal women than premenopausal women [4]. To better understand the molecular basis of sex-based differences, we present a systematic review and functional meta-analysis of publicly available genomic studies that analyze the differences in NAFLD progression between premenopausal women and men.

Despite the limitations of our approach (presence of studies with different sample sizes and types of platforms), meta-analysis techniques can integrate the selected studies. The individual results of the seven selected studies failed to identify any consistently different GO or KEGG terms between groups across all studies, a finding perhaps related to small sample sizes, underlying genetic and environmental variance in the source population, and methodological heterogeneity. Surprisingly, as shown in Fig. 3a, we found certain functions overrepresented in both groups in different studies. A meta-analysis, such as that carried out in this study, can help to improve the identification of consistent, relevant differences across studies, subject to the caveat that patients in each study may have other differences in clinical characteristics. A meta-analysis also increases the overall sample size and hence the strength of findings, which is of particular relevance to diseases such as NAFLD that displays high levels of heterogeneity. Indeed, systematic reviews and meta-analyses represent widely employed strategies in studying sex-based differences in other diseases [37–39].

Our functional meta-analysis identified a total of thirteen GO terms (Table 3) that displayed differences between men and premenopausal women using two different approaches—differentially expressed genes (nine terms) and differentially activated pathways (four terms). These terms broadly related to processes such as inflammation, cell binding, and the establishment of polarity during neural tube closure.

A functional meta-analysis of differentially expressed genes identified differences in nine significant GO terms. Among these, our results established the overrepresentation of the “*Vinculin binding”* (GO:0017166) in premenopausal women compared with men. The vinculin cytoplasmic actin-binding protein is enriched in focal adhesions and adherens junctions to contribute to junction stability [40] and plays a vital role as a regulator of apoptosis [41]. Of note, although “cellular adhesion” represents a broad set of processes rather than a specific biologic pathway, this term was previously reported as a GO term consistently enriched in male NASH patients [42]. Furthermore, Arendt et al. identified differentially expressed genes related to cell–cell adhesion (*ANXA2* and *MAG*) between NAFL and NASH patients [34].

Our analysis also identified sex-based differences related to the inflammatory response, specifically the expression of cytokine-related terms. We found “*Negative regulation of interleukin-6 secretion”* (GO:1900165) overrepresented in men and “*Cellular response to interleukin-2”* (GO:0071352) and “*Response to interleukin-2”* (GO:0070669) overrepresented in premenopausal women. These terms relate to interleukin 2 (IL-2) and interleukin 6 (IL-6), which displayed LOR values close to or above 0.5, thereby indicating a substantial difference between the groups. IL-2 is a known regulator of T cell responses, with low IL-2 levels regulating T cell central tolerance via the formation of regulatory T cells [43]. Higher IL-2 levels induce T cell proliferation and differentiation into memory T cells. IL-2 receptors are also expressed by B cells and cells of the innate immune system [44]. Interestingly, IL-2 signaling is deficient in many chronic liver diseases [45], and levels of soluble IL-2 receptor alpha were found to be higher in children with NASH and advanced fibrosis [46]. Our results suggest that low-level IL-2-induced immune tolerance may mitigate the risk of developing NASH in premenopausal women and raise the intriguing possibility of restoring low-level IL-2 signaling in men as a potential treatment for NAFLD.

IL-6 has been previously implicated in NASH, with increased hepatic expression correlating with disease severity and insulin resistance [47]. IL-6 also regulates hepatic regeneration [48] and represents a significant driver of hepatic carcinogenesis [49]. In NAFLD, IL-6 may enhance hepatic repair and regeneration; however, IL-6 may also promote insulin resistance and hepatocyte apoptosis, thus contributing to NASH development [48, 50]. Indeed IL-6 gene polymorphisms have been associated with increased susceptibility to chronic liver disease [51].

These results suggest that differences in immune mechanisms between men and women may represent one possible factor in the sex-differences observed in NAFLD. For example, the macrophage phenotype in male NAFLD mice presents as pro-inflammatory and pro-fibrotic, but pro-resolution and anti-fibrotic in female mice [52]. We hypothesize that women may suffer from a decreased immune response to lipotoxicity, whereas men may suffer from an impaired liver-healing response to chronic injury perhaps related to deficient IL-6 signaling. These ideas require further examination and validation in additional studies.

A recent study by Herrera-Marcos et al. detected sex-based differences in the control of the *Cidec/Fsp27β* gene in the liver, finding that gene expression correlated with the presence and density of liver lipid droplets in mice fed a high-fat diet [53]. Matsusue suggested a role for *Cidec/Fsp27β* as a neuronal activator in the steatotic liver [54]; therefore, the significant association with the function “*Negative regulation of neuron death”* (GO:1901215) could relate to the differences detected in this previous study. Upon restricting the gene ontology analysis to *Homo sapiens,* we found the components of the “*Negative regulation of neuron death”* pathway (GO: 1901215) as broad regulators of apoptosis. This finding may suggest an increase in liver cell apoptosis in men who develop advanced NASH at a younger age than women, as premenopausal women possess a level of protection. The observation that hepatocyte apoptosis represents a prominent feature of progressive NASH agrees with this hypothesis [55].

Interestingly, we discovered other significant GO terms that had not previously been associated with NAFLD. For instance, we found the overrepresentation of “*Establishment of planar polarity involved in neural tube closure”* (GO:0090177) and “*Regulation of establishment of planar polarity involved in neural tube closure”* (GO:0090178) in men with a LOR exceeding 0.5 (-0.514 and -0.523). Restricting analyses to *Homo sapiens* demonstrated that these GO terms relate to the Wnt and frizzled family of proteins. Wnt ligands and their cognate receptor frizzled represent critical determinants of liver development, zonation, regeneration, and responses to liver injury [56]. Their overrepresentation in male NASH patients could indicate the activation of disease repair pathways as a compensatory response to ongoing liver injury [57], as observed in preclinical models of NASH.

Our functional pathway meta-analysis identified four significant GO terms involved in cation transport: “*Cation transmembrane transport”* (GO:0098655), “*Metal ion transport”* (GO:0030001), “*Regulation of cation transmembrane transport”* (GO:1904062), and “*Inorganic cation transmembrane transport”* (GO:0098662). Previous studies have associated the alteration of pathways and functions related to cation transport with NAFLD [58]; however, information regarding relevance and significance remains scarce.

### Perspectives and significance

Our results support the existence of differences between men and premenopausal women in specific biological processes and molecular functions relevant to NAFLD. Given the well-known heterogeneity of NAFLD progression between men and women, this transcriptomic meta-analysis suggests the use of sex-specific pathophysiologically informed disease biomarkers. These findings may also impact disease diagnosis and prognosis and the design of clinical trials. For example, biological sex-driven differences may allow the enhanced classification of individuals with NAFLD and alter their inclusion in specific clinical trials. Our meta-analysis underscores the importance of recognizing and accounting for sex-related differences in the study of NAFLD pathobiology.

## Conclusions

While differences in clinical characteristics and pathogenic mechanisms in male and female NAFLD patients had been explored previously, details remained scarce. To this end, we selected seven studies suitable for functional meta-analysis and identified thirteen significant GO terms from these data. Of note, specific GO terms were previously identified to have relevance to NAFLD, which supports the notion that sex represents an important variable in understanding NAFLD pathobiology and heterogeneity. We identified novel functional terms that had not been linked to NAFLD previously; therefore, our study may open new avenues for further research. In conclusion, the meta-analysis of transcriptomic data represents a useful tool for the identification of sex-related differences in NAFL and NASH.

## Supplementary Information

**Additional file 1: Figure S1.** UpSet plots of our gene functional analysis for each individual study for GO terms overexpressed in premenopausal women or in men and KEGG pathways upregulated in premenopausal women or in men.

**Additional file 2: Figure S2.** Forest plots for significant GO terms from our functional gene meta-analysis.

**Additional file 3: Figure S3.** Forest plots of significant GO terms from our functional pathways meta-analysis.

**Additional file 4: Figure S4.** Funnel plots of the significant GO terms from our functional gene meta-analysis.

**Additional file 5: Figure S5.** Funnel plots of significant GO terms from our functional pathway meta-analysis.

**Additional file 6:** **Supplementary table.** Libraries version.

**Additional file 7:** **Supplementary file.** GEO Data.

## Data Availability

The large volume of data and results generated in this study are freely available through the metafun-NAFLD web tool: https://bioinfo.cipf.es/metafun-NAFLD. This study analyzed transcriptomic data available in the Gene Expression Omnibus database with accession numbers GSE48452, GSE61260, GSE66676, GSE83452, GSE89632, GSE126848, and GSE130970.

## Abbreviations

BH: Benjamini & Hochberg *p* value adjusting method
GEO: Gene Expression Omnibus
GO: Gene Ontology
GSA: Gene Set Analysis
KEGG: Kyoto Encyclopedia of Genes and Genomes
LOR: Log odds ratio
NAFLD: Non-alcoholic fatty liver disease
NAFL: Non-alcoholic steatosis
NASH: Non-alcoholic steatohepatitis
PCA: Principal Component Analysis
PAA: Pathway Activity Analysis
PRISMA: Preferred Reporting Items for Systematic Reviews and Meta-Analyses
RMA: Robust Multichip Average
