# Supplementary material for: Hepatic steatosis and steatohepatitis: a functional meta-analysis of sex-based differences in transcriptomic studies
Source: Biol Sex Differ. 2021 Mar 25;12:29. doi: 10.1186/s13293-021-00368-1 (PMC7995602; doi:10.1186/s13293-021-00368-1)
Supplement: Supplementary file 1 — Additional file 1: Figure S1. UpSet plots of our gene functional analysis for each individual study for GO terms overexpressed in premenopausal women or in men and KEGG pathways upregulated in premenopausal women or in men. [file 13293_2021_368_MOESM1_ESM.pdf]

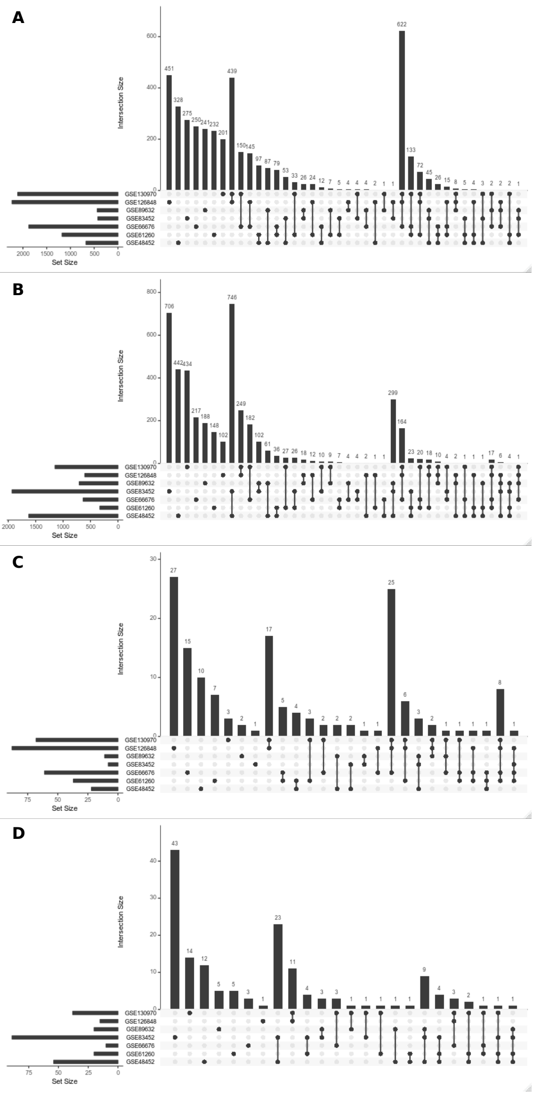

Supplementary Figure 1. UpSet plots of our gene functional analysis for each individual study for GO terms overexpressed in premenopausal women (A) or in men (B) and KEGG pathways upregulated in premenopausal women (C) or in men (D). Horizontal bars show the total number of significant elements in each study. The vertical bars below show the number of elements shared by the studies with a black dot joined by a line. For bars associated with only one point (i.e., not shared with any other study), the number of specific elements in that study is shown.
