# Supplementary material for: Hepatic steatosis and steatohepatitis: a functional meta-analysis of sex-based differences in transcriptomic studies
Source: Biol Sex Differ. 2021 Mar 25;12:29. doi: 10.1186/s13293-021-00368-1 (PMC7995602; doi:10.1186/s13293-021-00368-1)
Supplement: Supplementary file 2 — Additional file 2: Figure S2. Forest plots for significant GO terms from our functional gene meta-analysis. [file 13293_2021_368_MOESM2_ESM.pdf]

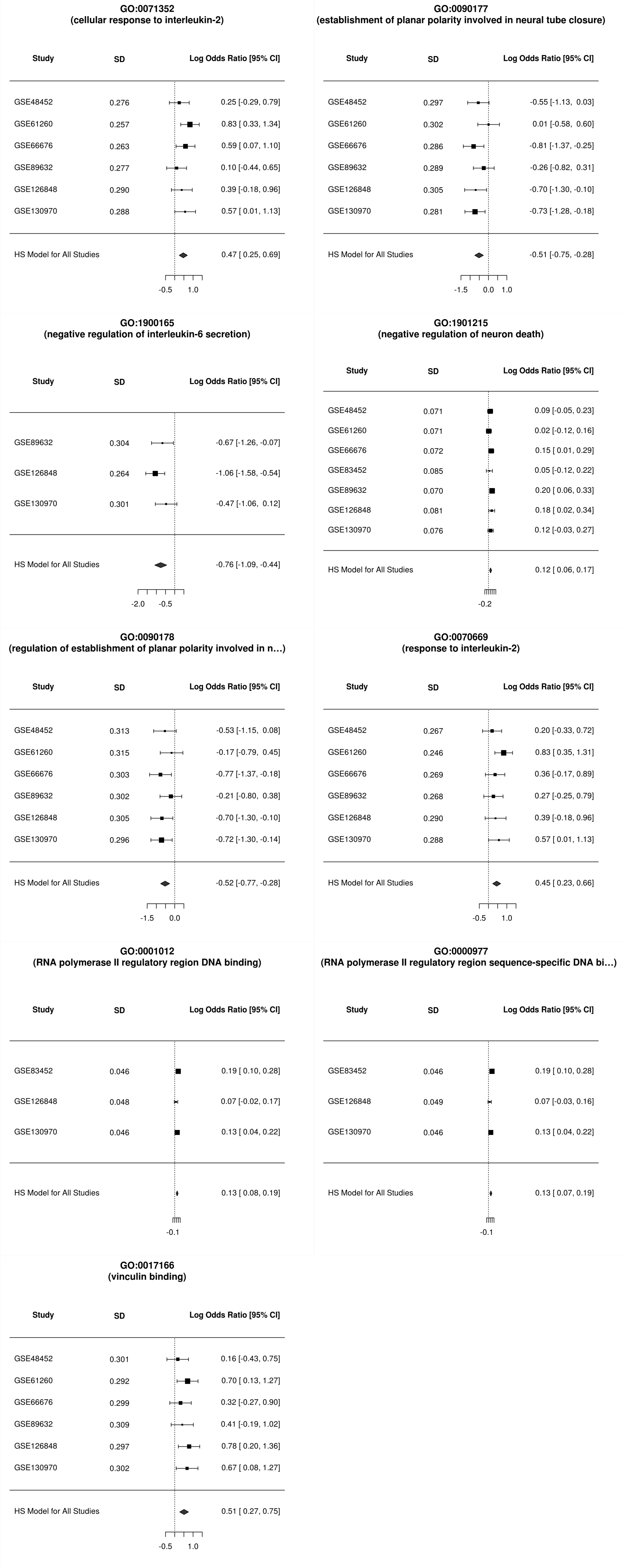

Supplementary Figure 2. Forest plots for significant GO terms from our functional gene meta-analysis.
